# Supplementary material for: Post-myocardial infarction heart failure dysregulates the bone vascular niche
Source: Nat Commun. 2021 Jun 25;12:3964. doi: 10.1038/s41467-021-24045-4 (PMC8233308; doi:10.1038/s41467-021-24045-4)
Supplement: Supplementary file 1 — Supplementary Information [file 41467_2021_24045_MOESM1_ESM.pdf]

a

Sex	Male
Age	12-weeks-old
EF (%) (Mean, SEM)	34.46, 2.25
Infarct size (%) (Mean, SEM)	52.75, 13.17

b

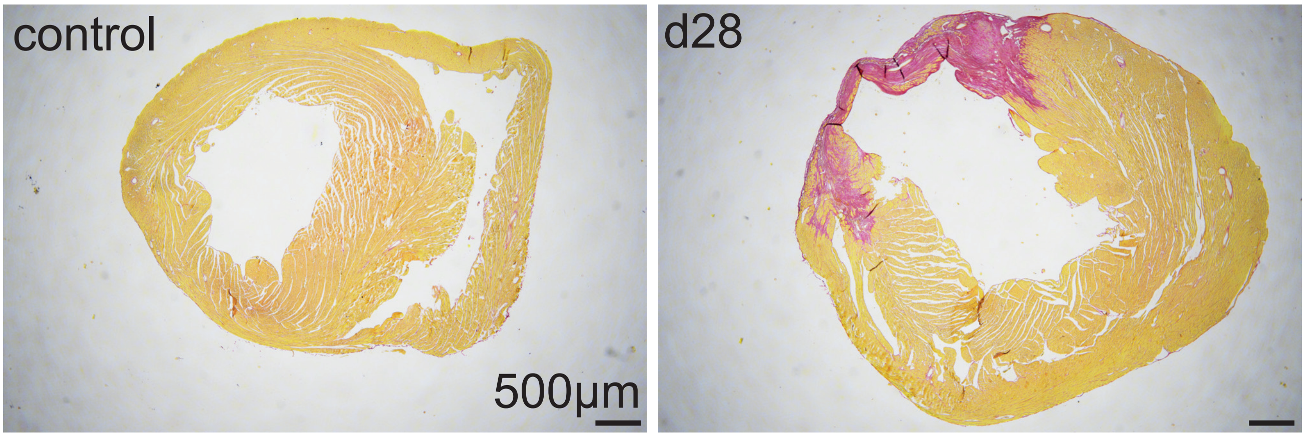

Hoffmann, Luxán, Abplanalp supfig. 1

Supplementary Figure 1. Representative analysis of myocardial infarction. (**a**) Table representing the sex, age, ejection fraction (EF; in %) in the first 24 h after coronary artery ligation and scar size (%) 28 days after the infarction. (**b**) Representative image of one control and infarct 28 days (d28) after infarct induction.

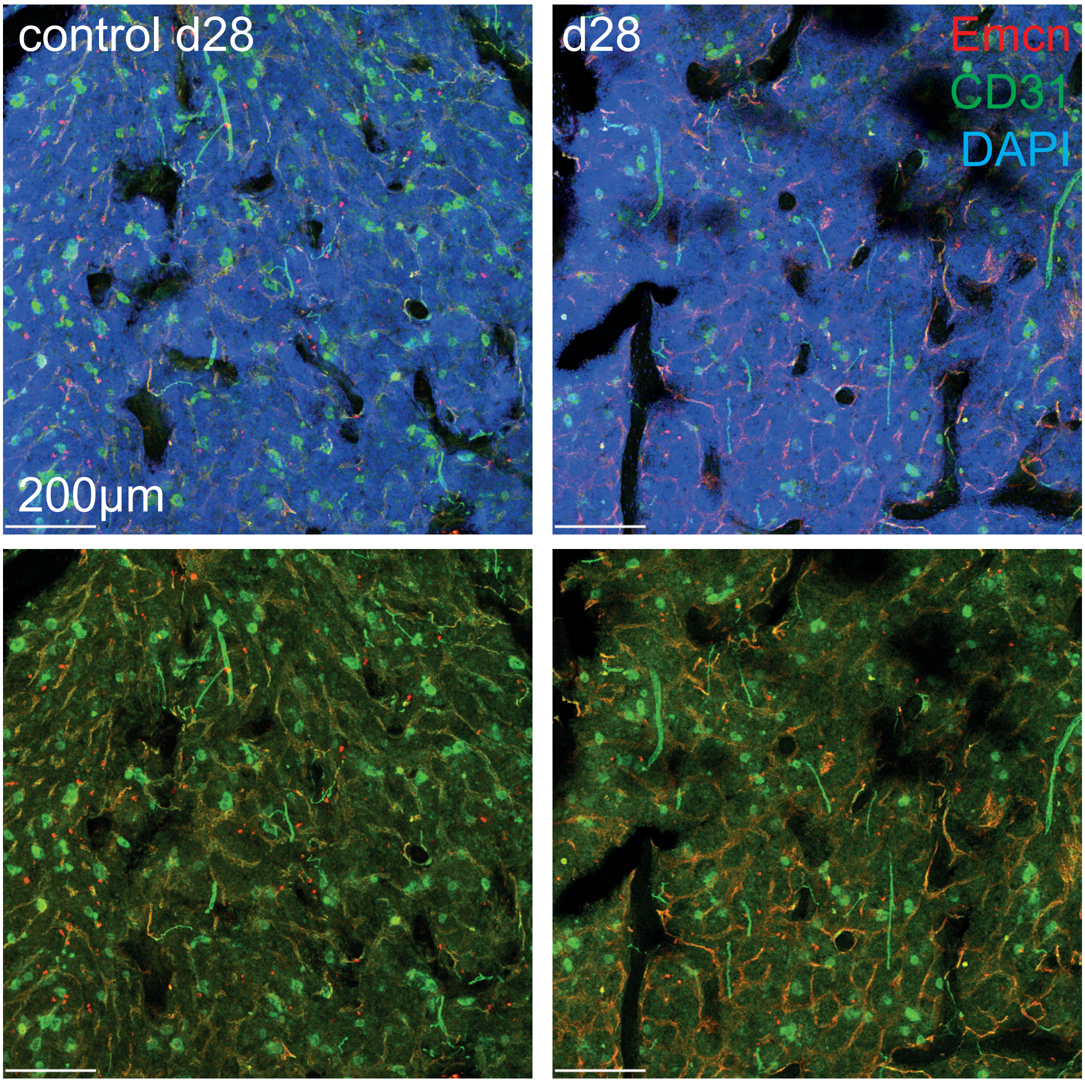

Hoffmann, Luxán, Abplanalp supfig. 2

Supplementary Figure 2. Type L cells in the diaphysis of the bone were not changed post MI. Representative images of the diaphysis area of bones shown in Figure 1.

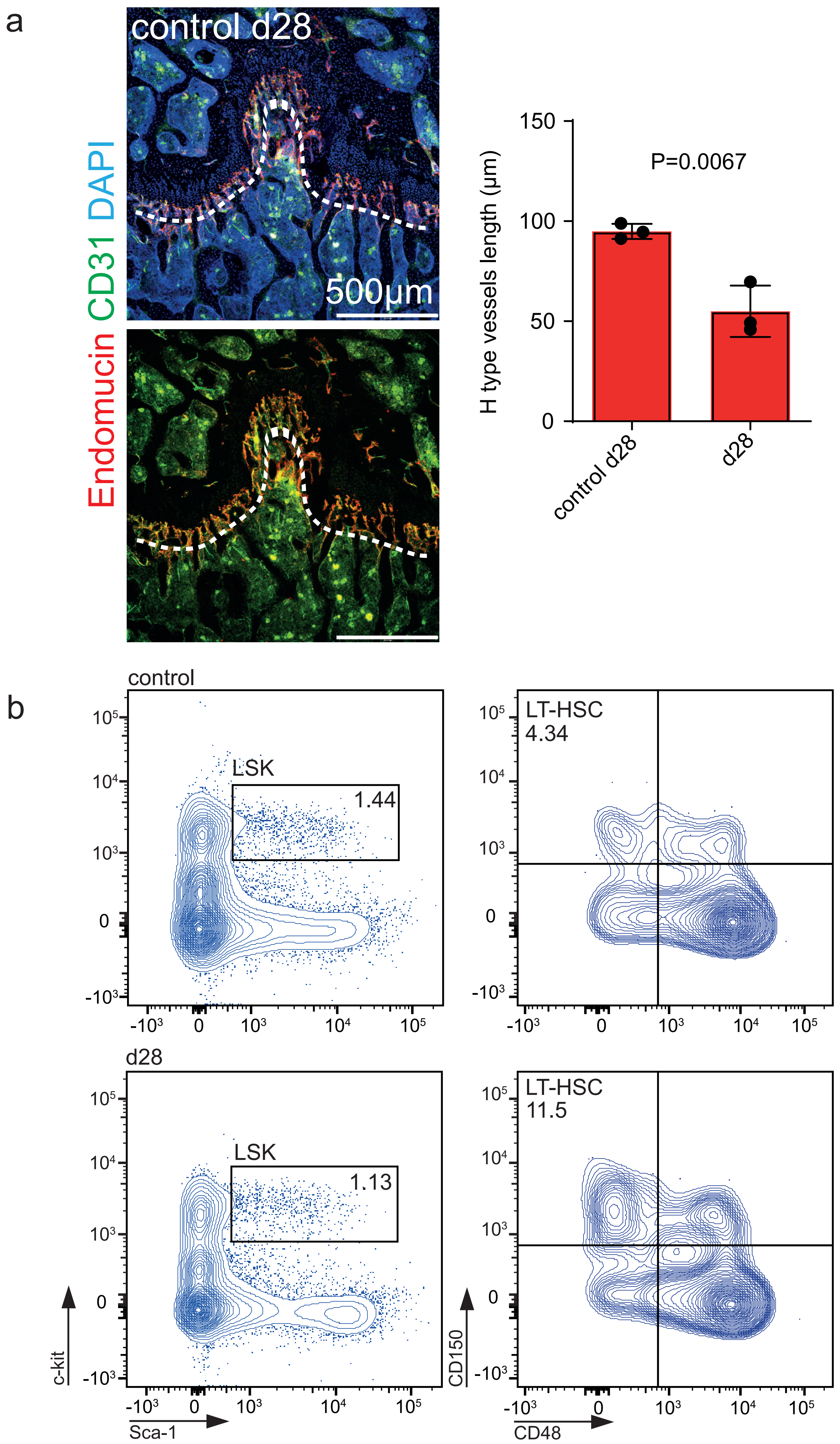

Hoffmann, Luxán, Abplanalp supfig. 3

Supplementary Figure 3. Reduction in type H endothelium upon MI is independent of age. (**a**) Flow cytometry analysis of femur bone marrow. Surgery was performed on 12-week-old animals. Gating strategy for hematopoietic stem cells. LSK, $Lin^{-}Sca\text{-}1^{+}c\text{-}kit^{+}$ cells; LT-HSC, long term hematopoietic stem cells. (**b**) Immunostaining of longitudinal sections through age-matched d28 control femur. Type H vessels are longer in the age-matched controls than in mice post MI. N=3. Data are shown as mean ± SEM. P-value was calculated by unpaired, two-tailed Student's *t*-test.

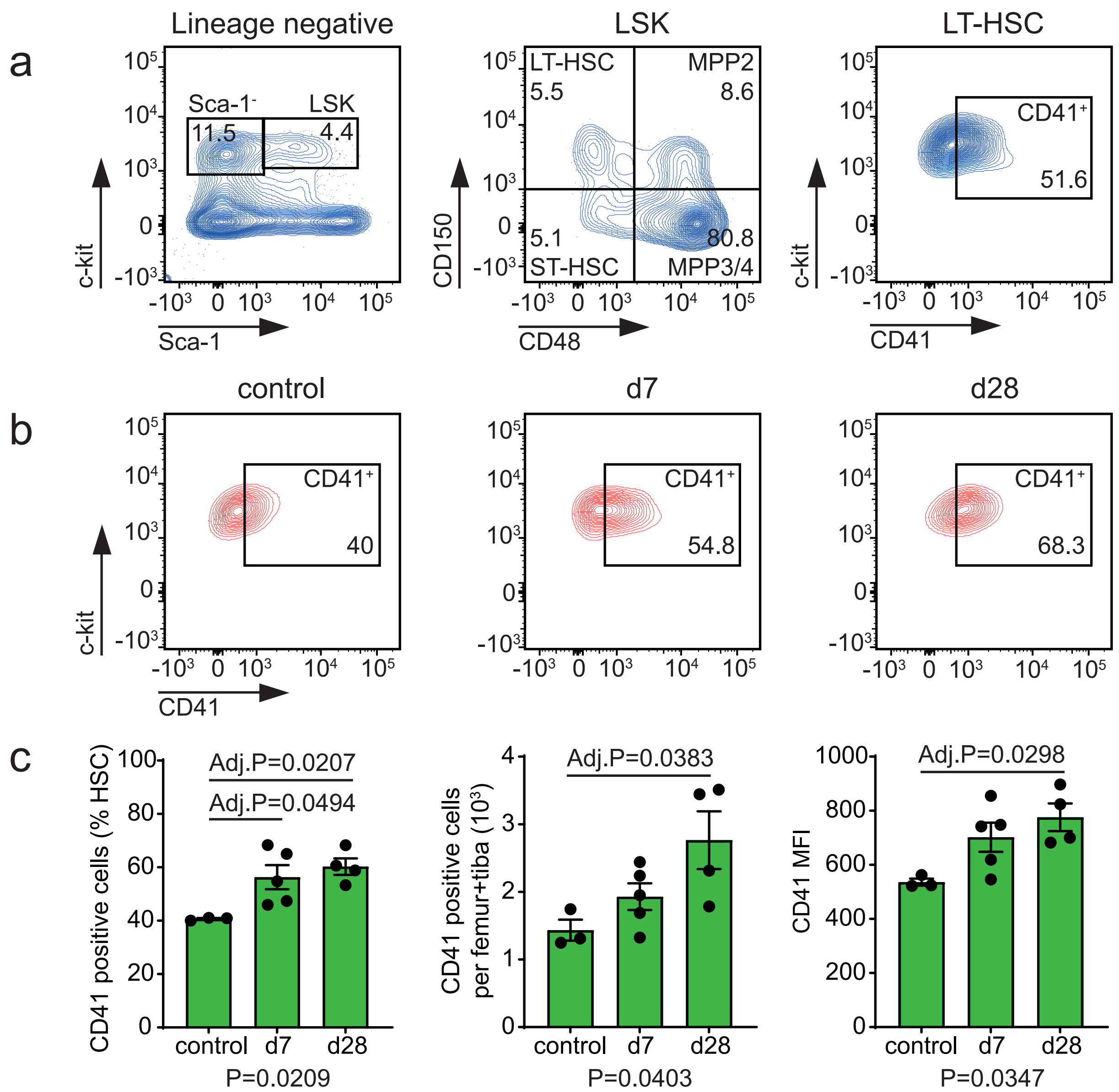

Hoffmann, Luxán, Abplanalp supfig. 4

Supplementary Figure 4. Detailed analysis of progenitor lineages confirm myeloid expansion. (**a**-**c**) Flow cytometry analysis of femur and tibia bone marrow 7 (d7) and 28 (d28) days after MI and control (d0). (**a**) Gating strategy for CD41 positive cells in the bone. LSK, $Lin^{-}Sca\text{-}1^{+}c\text{-}kit^{+}$ cells; MMP2, multipotent myeloid progenitor 2; MMP3/4, multipotent myeloid progenitor 3/4. ST-HSC, short term hematopoietic stem cells, LT-HSC, long term hematopoietic stem cells. (**b**) Representative flow chart of CD41 positive cells in femur and tibia bone marrow 7 (d7) and 28 (d28) days after MI and control (d0). (**c**) Quantification of CD41 positive cells represented by percentage of HSC cells (left), total numbers (middle), and CD41 mean fluorescence intensity (MFI) (right). N=3 for control, N=5 for d7 and N=4 for d28. Data are shown as mean ± SEM. P-value was calculated with ANOVA with Tukey's multiple comparison test. HSC, hematopoietic stem cells; MFI, mean fluorescence intensity.

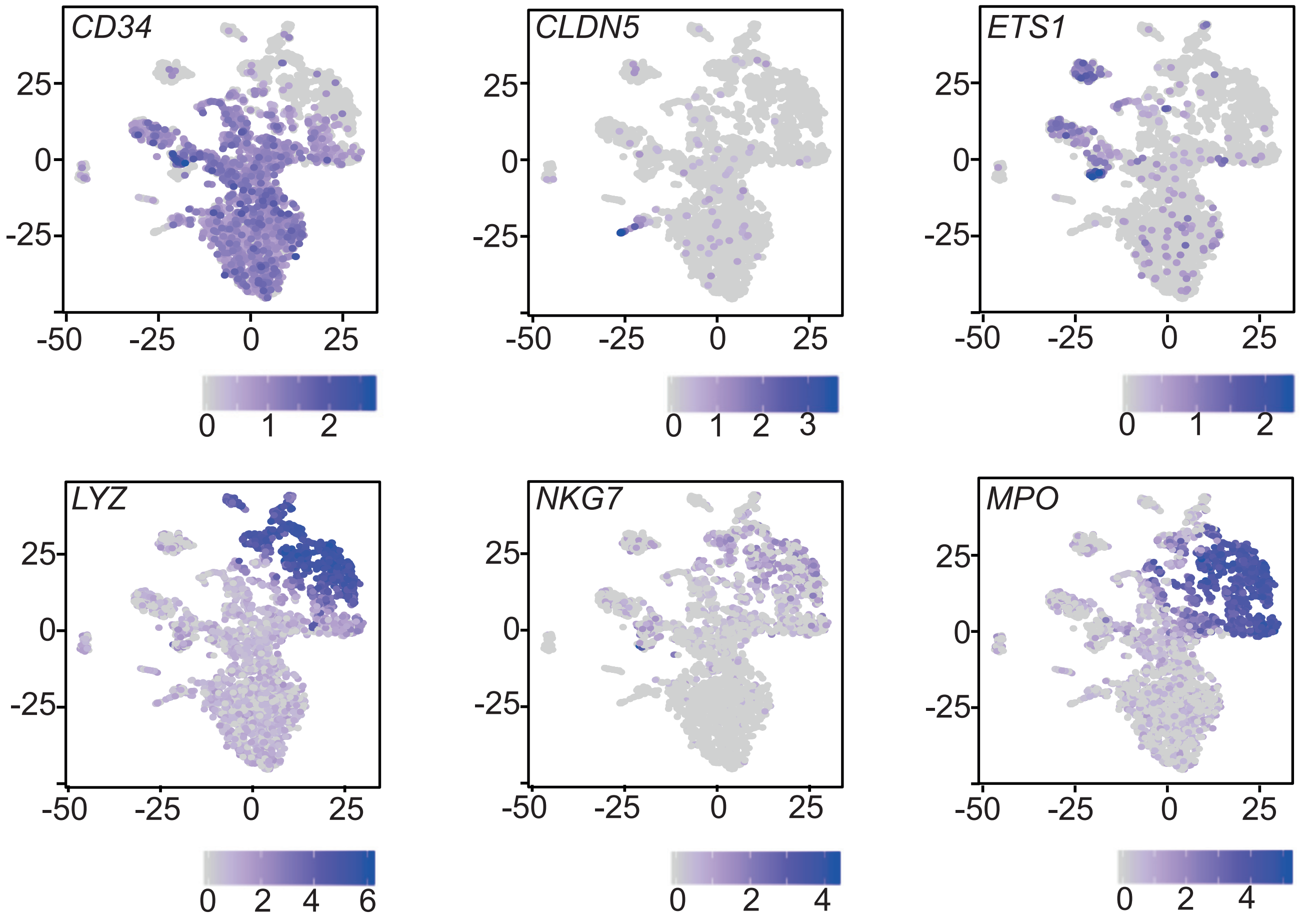

Hoffmann, Luxán, Abplanalp supfig. 5

Supplementary Figure 5. Expression of hallmark genes from scRNA-seq of bone marrow aspirates. Distribution of genes identifying groups of clusters shown in t-SNE format.

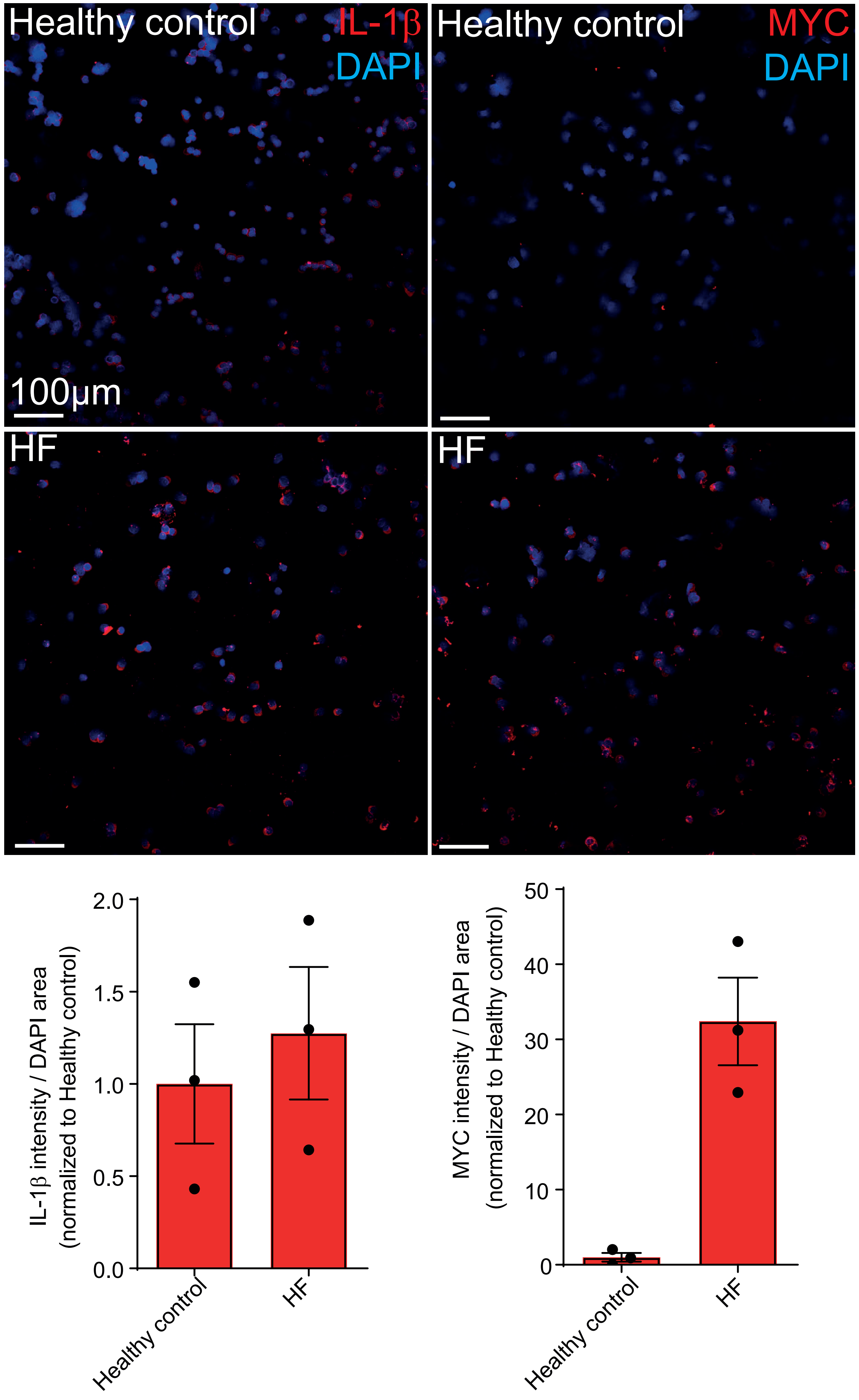

Hoffmann, Luxán, Abplanalp supfig. 6

Supplementary Figure 6. MYC and IL-1β are increased in patient bone marrow. Representative immunostaining of MACS-enriched endothelial cells from freshly bone marrow aspirates from healthy controls of heart failure patients (HF) Il1b or MYC are shown in red and DAPI in blue. Data are shown as mean ± SEM of the individual technical replicates per patient.

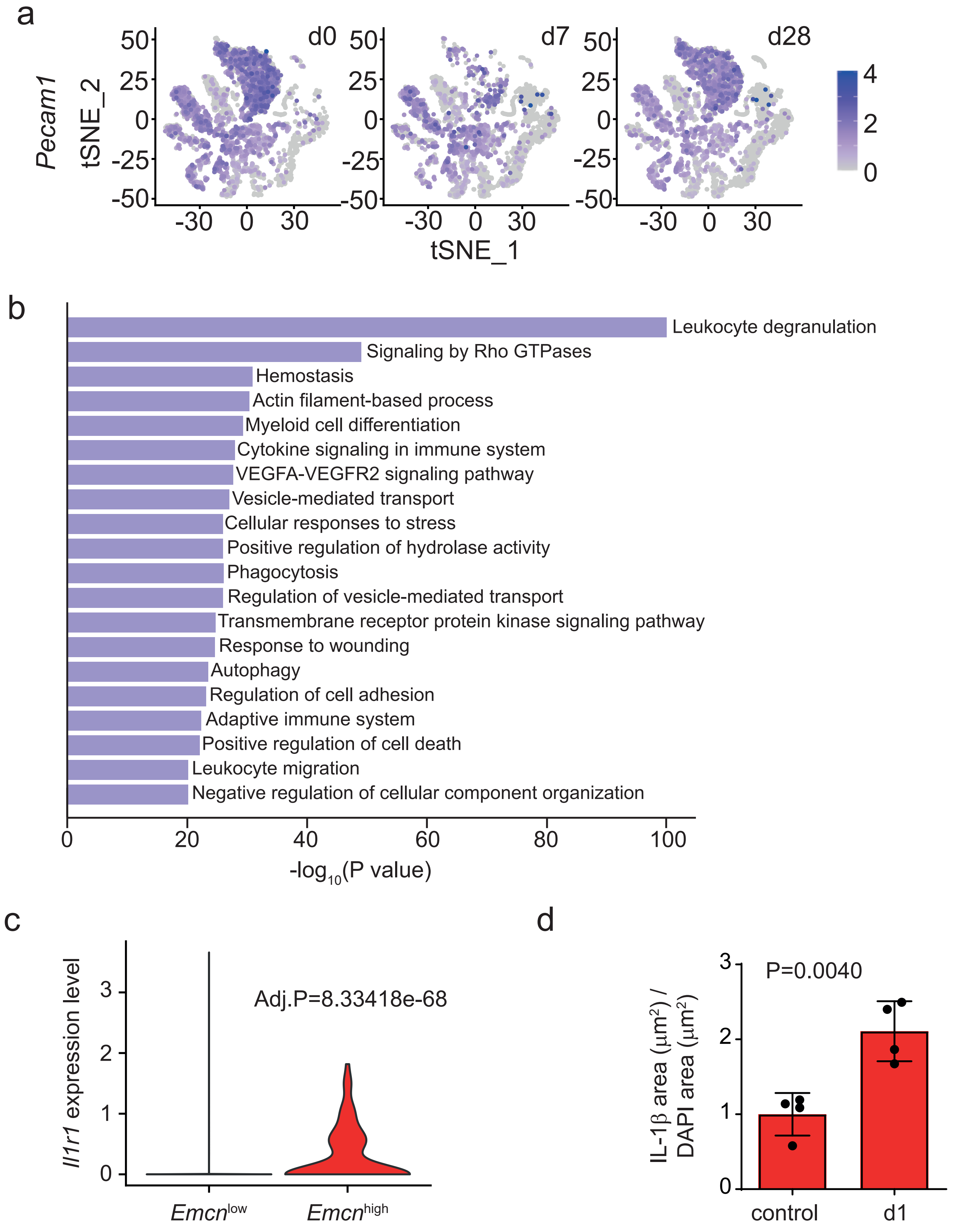

Hoffmann, Luxán, Abplanalp supfig. 7

Supplementary Figure 7. Inflammatory response in the bone after myocardial infarct. (**a**) scRNA-seq of mice bone marrow vascular niche with no infarction along with 7 (d7) and 28 days (d28) after myocardial infarction. Clustered cells from the three time points are displayed in t-SNE plots. *Pecam1*-expressing cells are coloured (**b**) Representation of the ten most significant upregulated terms at d28 revealed by gene ontology analysis when comparing d0 to d28. (**c**) Violin plot showing the expression of IL-1β receptor *Il1r1* is enriched in *Emcn*^{high} cells from single-cell-RNA-sequencing. P-value was calculated by *find markers* function in Seurat based on non-parametric Wilcoxon sum test with Bonferroni adjustment (**d**) Quantification of the immunohistochemistry shown in Figure 4e. N=4. Data are shown as mean ± SEM, *P*-value was calculated by unpaired, two-tailed Student's t test.

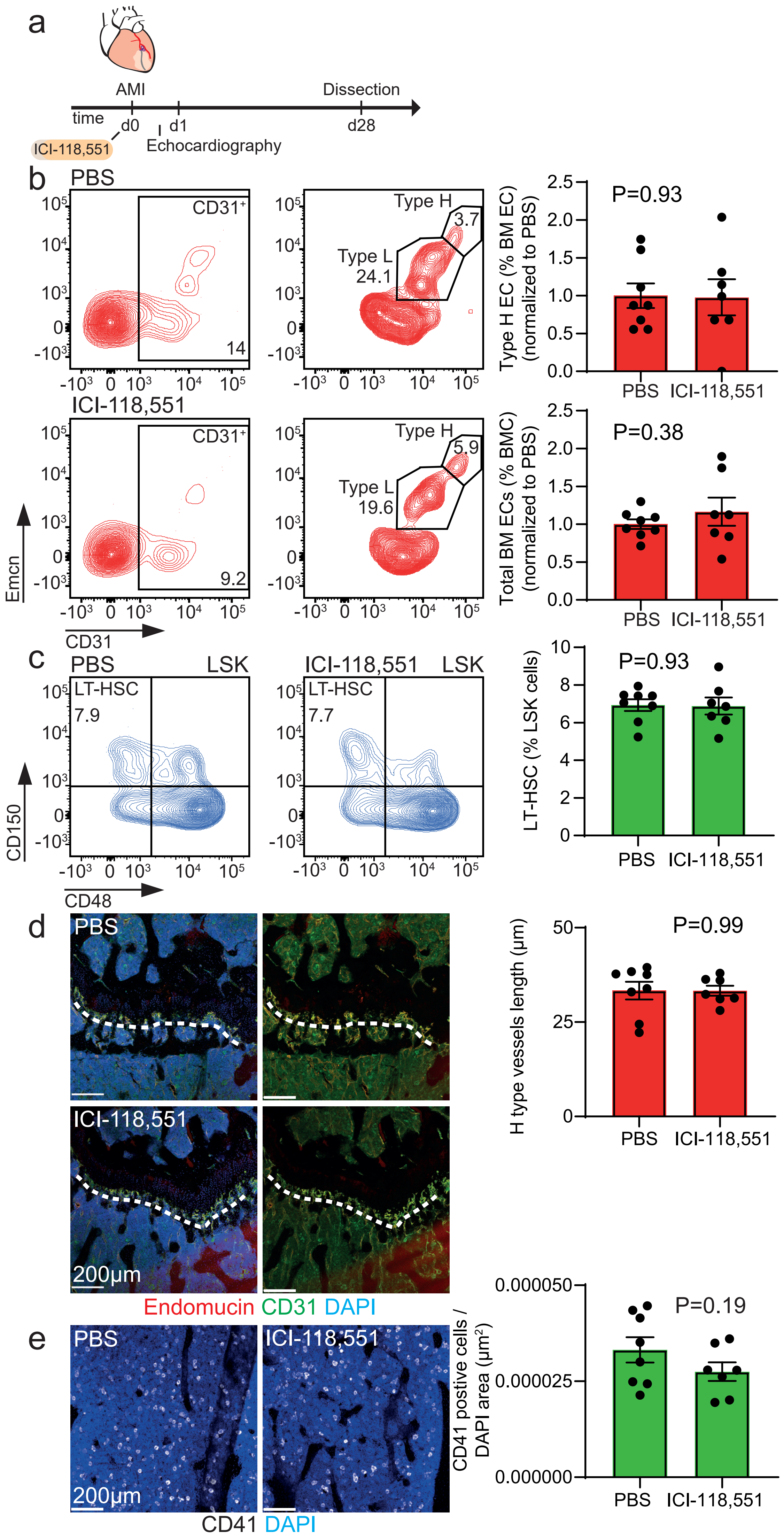

Hoffmann, Luxán, Abplanalp supfig. 8

Supplementary Figure 8. Blocking β2AR signalling does not protect the bone vascular niche after myocardial infarct. (**a**) Schematic of the experimental design. (**b, c**) Flow cytometry analysis of femur and tibia. (**b**) Type H endothelial cell number is not changed upon β2AR (left, gating strategy; right, quantification), N=8 for PBS and N=7 for ICI-118,551. Data are shown as mean ± SEM. P-value was calculated by unpaired, two-tailed Student's *t*-test. (**c**) Analysis of haematopoietic stem and progenitor subsets showing no significant changes in numbers of progenitors after ICI-118,551 treatment. (left, gating strategy; right, quantification), N=8 for PBS and N=7 for ICI-118,551. Data are shown as mean ± SEM. P-value was calculated by unpaired, two-tailed Student's t test. (**d, e**) Immunostaining of longitudinal femur sections. (**d**) The length of type H vessels (dashed line) is not change upon ICI-118,551 treatment 28 days after MI. N=6 for both groups. Data are shown as mean ± SEM. P-value was calculated by unpaired, two-tailed Student's *t*-test. (**e**) Myeloid progenitor cell number is not changed in the bone marrow of ICI-118,551 treated animals after infarct. N=6 for both groups. Data are shown as mean ± SEM. P-value was calculated by unpaired, two-tailed Student's *t*-test.

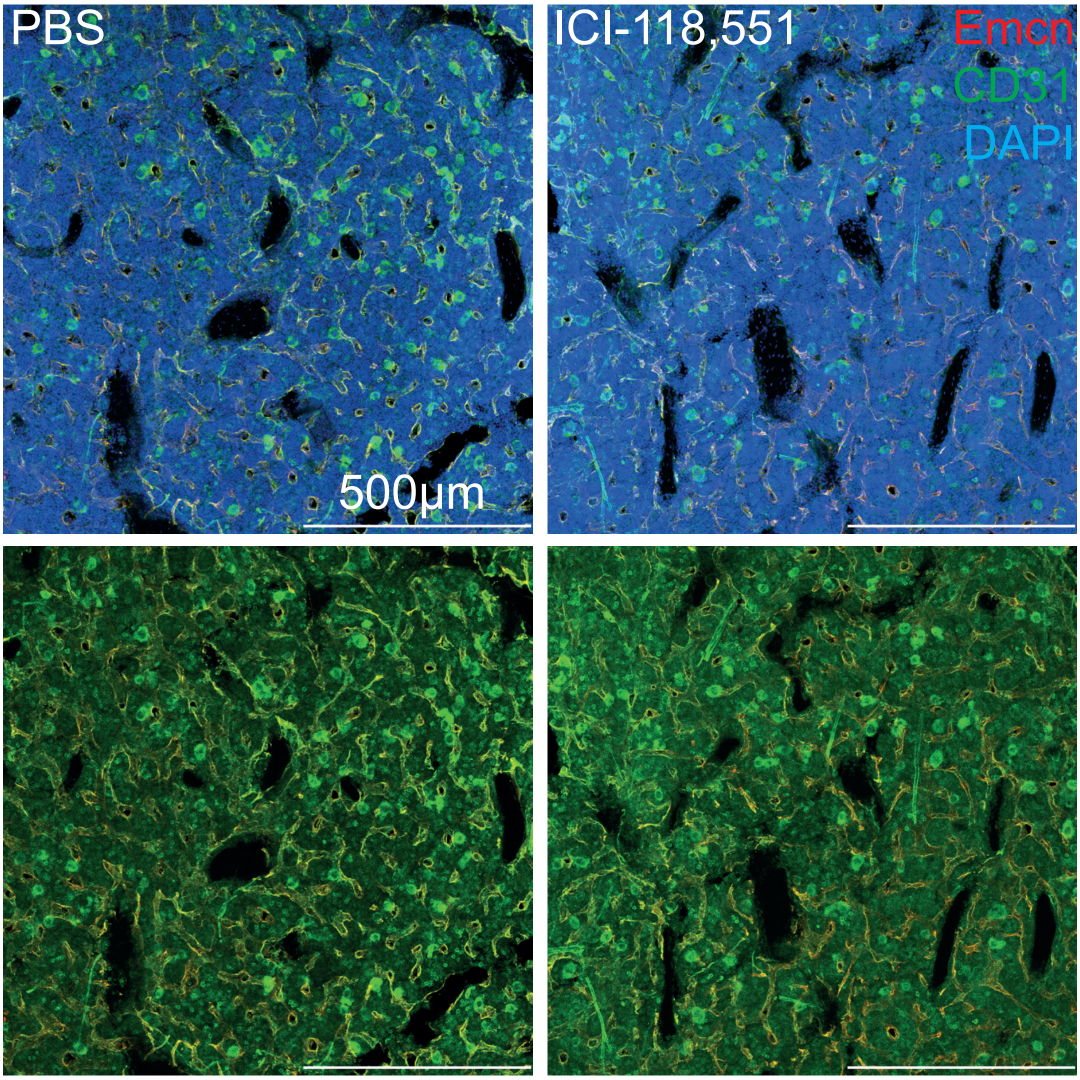

Hoffmann, Luxán, Abplanalp supfig. 9

Supplementary Figure 9. Vessels in the diaphysis region 28 days after myocardial infarct in ICI-118,551 treated mice. Representative images of the diaphysis area of bones shown in Supplementary Figure 8. N=6 for both groups.

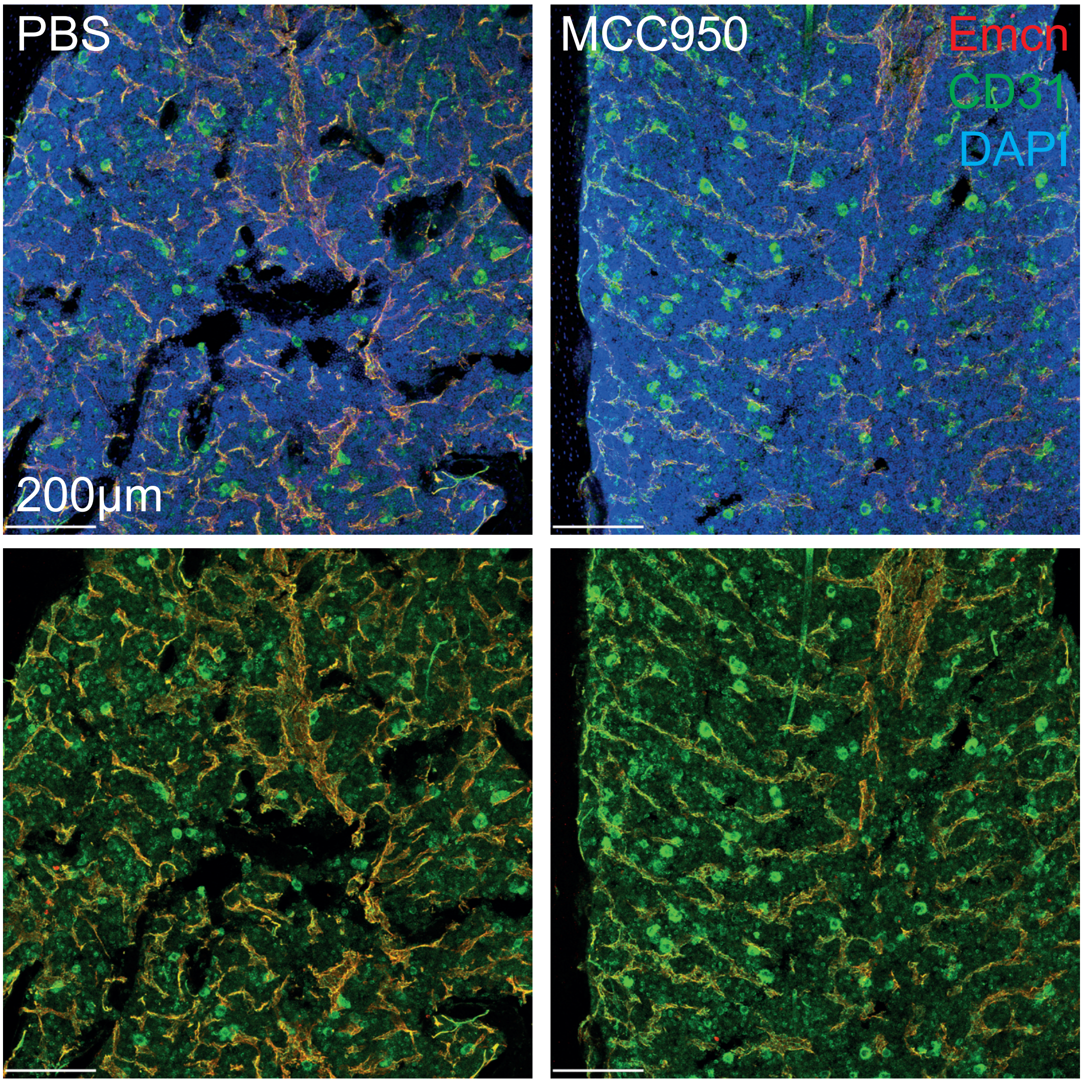

Hoffmann, Luxán, Abplanalp supfig. 10

Supplementary Figure 10. Vessels in the diaphysis region 28 days after myocardial infarct in MCC950 treated mice. Representative images of the diaphysis area of bones shown in Figure 4. N=7 for PBS and N=6 for MCC950.

Supplemental Table 1. Baseline characteristics of the CHF patient and control study cohort

	CHF (FACS)	CHF (scRNA-Seq)	CTRL (FACS)	CTRL (scRNA-Seq)
n	18	1	8	1
Age (years)	60 [IQR 54-71]	43	31 [IQR 27-37]	35
Sex (male/female)	17/1	1/0	4/4	1/0
NYHA class [I/II/III/IV (n)]	0/9/9/0	0/1/0/0	8/0/0/0	1/0/0/0
Hypertension [n (%)]	13 (72)	1	0 (0)	0
Hypercholesterolemia [(%)]	12 (67)	1	0 (0)	0
Diabetes [n (%)]	4 (22)	0	0 (0)	0
Smoking [n (%)]	14 (78)	1	3 (38)	1
Coronary artery disease [1/2/3 vessel disease (n)]	4/6/8	0/1/0	0/0/0	0/0/0
Time from last MI (months)	60 [IQR 15-145]	7	N/A	N/A
Left ventricular EF (%)	28 [IQR 22-35]	23	-	-
ICD or CRT-D (%)	15 (83)	1	0 (0)	0
ACEi or ATRB (%)	17 (94)	1	0 (0)	0
Beta-blocker (%)	16 (89)	1	0 (0)	0
Diuretic (%)	16 (89)	1	0 (0)	0
Aldosterone antagonist	15 (83)	1	0 (0)	0
Statin	18 (100)	1	0 (0)	0

CHF - chronic heart failure
MI – myocardial Infarction
EF – ejection fraction
ICD – implantable cardioverter defibrillator
ACEi – angiotensin-converting enzyme inhibitor
ATRB - Angiotensin II receptor blocker

Supplementary Table 2. RT-qPCR primers

Murine MYC forward	CGGATTCTCTGCTCTCCTCG
Murine MYC reverse	TCATCTTCTTGTTCCTCCTCAGA
Human MYC forward	AGCTGCTTAGACGCTGGATTTT
Human MYC reverse	TCGAGGTCATAGTTCCTGTTGG
Murine Rplp0 forward	TTTGACAACGGCAGCATTTA
Murine Rplp0 reverse	CCGATCTGCAGACACACACT
Human Rplp0 forward	GGCGACCTGGAAGTCCAACT
Human Rplp0 reverse	CCATCAGCACCACAGCCTTC
