# Supplementary material for: Post-myocardial infarction heart failure dysregulates the bone vascular niche
Source: Nat Commun. 2021 Jun 25;12:3964. doi: 10.1038/s41467-021-24045-4 (PMC8233308; doi:10.1038/s41467-021-24045-4)
Supplement: Supplementary file 3 — Reporting Summary [file 41467_2021_24045_MOESM3_ESM.pdf]

nature research

Corresponding author(s): Stefanie Dimmeler

Last updated by author(s): Apr 20, 2021

Reporting Summary

Nature Research wishes to improve the reproducibility of the work that we publish. This form provides structure for consistency and transparency in reporting. For further information on Nature Research policies, see Authors & Referees and the Editorial Policy Checklist.

Statistics

For all statistical analyses, confirm that the following items are present in the figure legend, table legend, main text, or Methods section.

n/a	Confirmed	
☐	☒	The exact sample size (n) for each experimental group/condition, given as a discrete number and unit of measurement
☐	☒	A statement on whether measurements were taken from distinct samples or whether the same sample was measured repeatedly
☐	☒	The statistical test(s) used AND whether they are one- or two-sided<br>*Only common tests should be described solely by name; describe more complex techniques in the Methods section.*
☐	☒	A description of all covariates tested
☐	☒	A description of any assumptions or corrections, such as tests of normality and adjustment for multiple comparisons
☐	☒	A full description of the statistical parameters including central tendency (e.g. means) or other basic estimates (e.g. regression coefficient) AND variation (e.g. standard deviation) or associated estimates of uncertainty (e.g. confidence intervals)
☐	☒	For null hypothesis testing, the test statistic (e.g. F, t, r) with confidence intervals, effect sizes, degrees of freedom and P value noted<br>*Give P values as exact values whenever suitable.*
☒	☐	For Bayesian analysis, information on the choice of priors and Markov chain Monte Carlo settings
☒	☐	For hierarchical and complex designs, identification of the appropriate level for tests and full reporting of outcomes
☒	☐	Estimates of effect sizes (e.g. Cohen's d, Pearson's r), indicating how they were calculated

Our web collection on statistics for biologists contains articles on many of the points above.

Software and code

Policy information about availability of computer code

Data collection	Volocity 6.5, Leica Application Suite X 2.0.1.14392, FACS Diva 6.0.
Data analysis	Volocity 6.5, Graphpad 9, Seurat 3.1.5, Monocle 2, FlowJo (Version 10, FlowJo LLC), Metascape 3.5, DAVID Bioinformatics Resources 6.7, Cell Ranger Single Cell Software Suite 2.

For manuscripts utilizing custom algorithms or software that are central to the research but not yet described in published literature, software must be made available to editors/reviewers. We strongly encourage code deposition in a community repository (e.g. GitHub). See the Nature Research guidelines for submitting code & software for further information.

Data

Policy information about availability of data

All manuscripts must include a data availability statement. This statement should provide the following information, where applicable:

- Accession codes, unique identifiers, or web links for publicly available datasets
- A list of figures that have associated raw data
- A description of any restrictions on data availability

The single-cell RNA–seq data sets generated in this study are available at Array Express (https://www.ebi.ac.uk/arrayexpress) with the following accession numbers: E-MTAB-10432 and E-MTAB-10448. All other data are included within the article, source data, and supplementary data can be made available upon request. Source data are provided with this paper.

Field-specific reporting

Please select the one below that is the best fit for your research. If you are not sure, read the appropriate sections before making your selection.

☒ Life sciences ☐ Behavioural & social sciences ☐ Ecological, evolutionary & environmental sciences

For a reference copy of the document with all sections, see nature.com/documents/nr-reporting-summary-flat.pdf

Life sciences study design

All studies must disclose on these points even when the disclosure is negative.

Sample size	Required sample sizes for in vivo experiments were estimated using power-calculation (p=0.8). For in vitro experiments sample number was defined according to previous experience and reproducibility of the results across several independent experiments.
Data exclusions	No data was excluded
Replication	For all in vivo experiments, the number of replicates is given in the respective figure legends. Individual values are shown in each figure. In vitro experiments were generally performed in at least 2 independent experiments to ensure reproducibility of the data
Randomization	Animal have been randomly assigned to treatment cohorts. Cells have been randomly allocated to respective groups.
Blinding	Researchers were blind to mouse genotype, treatment or patient status for quantification. For scRNAseq experiments scientists were blinded during library generation and data aggregation, and were unblinded for final statistical analysis.

Reporting for specific materials, systems and methods

We require information from authors about some types of materials, experimental systems and methods used in many studies. Here, indicate whether each material, system or method listed is relevant to your study. If you are not sure if a list item applies to your research, read the appropriate section before selecting a response.

Materials & experimental systems

n/a	Involved in the study	
☐	☒	Antibodies
☒	☐	Eukaryotic cell lines
☒	☐	Palaeontology
☐	☒	Animals and other organisms
☐	☒	Human research participants
☐	☒	Clinical data

Methods

n/a	Involved in the study	
☒	☐	ChIP-seq
☐	☒	Flow cytometry
☒	☐	MRI-based neuroimaging

Antibodies

Antibodies used

All antibodies are commercially purchased and detailed information for them is provided in the Methods section.

Mouse flow cytometry:
HSC Panel: FITC-conjugated Lineage cocktail (133301; Biolegend; clones 145-2C11, RB6-8C5, M1/70, RA3-6B2, Ter-119), BV421-conjugated c-kit (105827; Biolegend; clone 2B8), PE-Cy7- conjugated Ly-6A (122513; Biolegend; clone E13-161.7), APC-conjugated CD48 (103411; Biolegend; clone HM48-1), PE-conjugated CD150 (115903; Biolegend; clone TC15-12F12.2), BV-510 conjugated CD41 (133923; Biolegend; clone MWReg30) and APC-Cy7-conjugated CD16/32 (101328; Biolegend; clone 93).
EC Panel: FITC-conjugated Ter119 (116206; Biolegend), rabbit anti Ephrin-B2 (ab131536; abcam), eFluor 660-conjugated anti Endomucin (50-5851-82; Thermo fisher scientific; clone V.7C7), BV510-conjugatetd CD45 (103137; Biolegend; clone 30-F11) and PE-Cy7-conjugated CD31 (102418; Biolegend; clone 390). secondary antibodies (BV421-conjugated donkey anti-rabbit (406410; Biolegend)

Human bone marrow flow cytometry: BV-421-conjugated CD31 (303124; Biolegend; clone WM59), APC-Cy7-conjugated CD45 (368516; Biolegend; clone 2D1), FITC-conjugated Lineage cocktail 4 (562722; BD; clones RPA-2.10, HIT3a, RPA-T4, M-T701, HIT8a, B159, GA-R2 (HIR2)), APC-conjugated AC133 (130-090-826; Miltenyi Biotec), PE-Cy7-conjugated CD34 (343516; Biolegend; clone 581) and biotin-conjugated Endomucin (ab45772; abcam; clone TX18). Secondary antibody: PE-conjugated Streptavidin (405204; Biolegend).

Human bone marrow immunocytochemistry: Mouse anti-c-Myc (MA1-980, Thermofisher) and Biotin anti-IL-1β (511703, Biolegend). Goat anti-mouse Alexa Fluor 555 (A-21425, Life Technologies) and Streptavidin Alexa Fluor 555 (S21381, Life Technologies).

Cryo-section immunostaining: Goat anti CD31 Alexa Fluor 488 conjugated (FAB3628G, R&D), rat anti Endomucin (sc-65495,

Santa Cruz), rat anti CD41 Alexa Fluor 647 conjugated (133934, Biolegend), and mouse anti IL1 (12242, Cell Signalling). Donkey anti rat Alexa Fluor 594 (A21209, Life Technologies) and donkey anti mouse Alexa Fluor 647 (A31571, Life Technologies).

Validation

Primary antibodies were validated by the manufacturer and confirmed by specific labeling of target molecules or cell types.

FITC-conjugated Lineage cocktail (133301; Biolegend; clones 145-2C11, RB6-8C5, M1/70, RA3-6B2, Ter-119) (https://www.biolegend.com/en-us/products/fitc-anti-mouse-lineage-cocktail-with-isotype-ctrl-5803)
BV421- conjugated c-kit (105827; Biolegend; clone 2B8) (https://www.biolegend.com/de-de/products/brilliant-violet-421-anti-mouse-cd117-c-kit-antibody-7157)
PE-Cy7- conjugated Ly-6A (122513; Biolegend; clone E13-161.7) (https://www.biolegend.com/en-ie/products/pe-cyanine7-anti-mouse-ly-6a-e-sca-1-antibody-3898)
APC-conjugated CD48 (103411; Biolegend; clone HM48-1) (https://www.biolegend.com/nl-be/products/apc-anti-mouse-cd48-antibody-3622)
PE-conjugated CD150 (115903; Biolegend; clone TC15-12F12.2) (https://www.biolegend.com/en-gb/sean-tuckers-tests/pe-anti-mouse-cd150-slam-antibody-1369)
BV-510 conjugated CD41 (133923; Biolegend; clone MWReg30) (https://www.biolegend.com/en-us/products/brilliant-violet-510-anti-mouse-cd41-antibody-10072)
APC-Cy7-conjugated CD16/32 (101328; Biolegend; clone 93) (https://www.biolegend.com/de-de/search-results/apc-cyanine7-anti-mouse-cd1632-antibody-6283)
FITC-conjugated Ter119 (116206; Biolegend) (https://www.biolegend.com/en-ie/products/fitc-anti-mouse-ter-119-erythroid-cells-antibody-1865)
rabbit anti Ephrin-B2 (ab131536; abcam) (https://www.abcam.com/ephrin-b2-antibody-ab131536.html)
eFluor 660-conjugated anti Endomucin (50-5851-82, Thermo fisher scientific; clone V.7C7) (https://www.thermofisher.com/antibody/product/Endomucin-Antibody-clone-eBioV-7C7-V-7C7-Monoclonal/50-5851-82)
BV510-conjugatetd CD45 (103137; Biolegend; clone 30-F11) (https://www.biolegend.com/de-de/search-results/brilliant-violet-510-anti-mouse-cd45-antibody-7995)
PE-Cy7-conjugated CD31 (102418; Biolegend; clone 390) (https://www.biolegend.com/de-de/products/pe-cyanine7-anti-mouse-cd31-antibody-3942)
BV-421-conjugated CD31 (303124; Biolegend; clone WM59) (https://www.biolegend.com/de-de/products/brilliant-violet-421-anti-human-cd31-antibody-8588)
APC-Cy7-conjugated CD45 (368516; Biolegend; clone 2D1) (https://www.biolegend.com/de-de/products/apc-cyanine7-anti-human-cd45-antibody-12400)
FITC-conjugated Lineage cocktail 4 (562722; BD; clones RPA-2.10, HIT3a, RPA-T4, M-T701, HIT8a, B159, GA-R2 (HIR2)) (https://www.bdbiosciences.com/us/applications/research/stem-cell-research/stem-cell-kits-and-cocktails/human/human-lineage-cocktail-4-lin-4-cd2-cd3-cd4-cd7-cd8-cd10-cd11b-cd14-cd19-cd20-cd56-cd235a/p/562722)
APC-conjugated AC133 (130-090-826; Miltenyi Biotec) (https://www.miltenyibiotec.com/DE-en/products/cd133-1-antibody-anti-human-ac133.html)
PE-Cy7-conjugated CD34 (343516; Biolegend; clone 581) (https://www.biolegend.com/de-de/search-results/pe-cyanine7-anti-human-cd34-antibody-6160)
biotin-conjugated Endomucin (ab45772; abcam; clone TX18)
Mouse anti-c-Myc (MA1-980, Thermofisher) (https://www.thermofisher.com/antibody/product/c-Myc-Antibody-clone-9E10-Monoclonal/MA1-980)
Biotin anti-IL-1β (511703, Biolegend) (https://www.biolegend.com/en-gb/products/biotin-anti-human-il-1beta-antibody-4416)
Goat anti CD31 Alexa Fluor 488 conjugated (FAB3628G, R&D) (https://www.rndsystems.com/products/mouse-rat-cd31-pecam-1-alexa-fluor-488-conjugated-antibody_fab3628g)
rat anti Endomucin (sc-65495, Santa Cruz) (https://www.scbt.com/p/endomucin-antibody-v-7c7)
rat anti CD41 Alexa Fluor 647 conjugated (133934, Biolegend) (https://www.biolegend.com/fr-ch/search-results/alexa-fluor-647-anti-mouse-cd41-antibody-16506)
mouse anti IL1-beta (12242, Cell Signalling) (https://www.cellsignal.de/products/primary-antibodies/il-1b-3a6-mouse-mab/12242)

Secondary antibodies have been tested in our experimental conditions to rule out unspecific reactivity.

Animals and other organisms

Policy information about studies involving animals; ARRIVE guidelines recommended for reporting animal research

Laboratory animals	A full list of the mouse strains used is detailed in the Methods Section. No other laboratory animals were used. 12 weeks-old male C57Bl/6J mice and 8-weeks-old MycEC-OE mice were used in this study
Wild animals	No wild animals were used.
Field-collected samples	No field-collected samples were used.
Ethics oversight	All animal procedures were performed according to relevant laws and institutional guidelines, were approved by local animal ethics Tierschutzbeauftragte from the Goethe University Frankfurt and were conducted with permissions FU/1218 and FU/1222 granted by the Regierungspräsidium Darmstadt of Hessen.

Note that full information on the approval of the study protocol must also be provided in the manuscript.

Human research participants

Policy information about studies involving human research participants

Population characteristics	Bone marrow aspirates were obtained from healthy volunteers without any evidence of coronary artery disease in their history (N=8; 50% male; median age 31 years [IQR 27-37], including 1 volunteer for scRNA-Seq experiments, male, age 35 years) as well from patients suffering from post-infarct heart failure with severely reduced left ventricular function (N=18; 94% male; median age 60 years [IQR 54-71]; and 1 patient for scRNA-Seq, male, age 43 years), undergoing intracoronary infusion of bone marrow mononuclear cells within the REPEAT trial.
Recruitment	We enrolled 19 consecutive REPEAT trial patients. Heathy controls were recruited on a voluntary basis according to the ethics review board of the Goethe University in Frankfurt, Germany (Approval No. 160/15 for healthy controls and the study consent for REPEAT trial).<br>Healthy volunteers were usually recruited among younger employees on-site without any known cardiovascular disease. While self-selection bias applies, it appears unlikely to have significantly affected the results of the present study.
Ethics oversight	The ethics review board of the Goethe University in Frankfurt, Germany, approved the protocols (Approval No. 160/15 for healthy controls and the study consent for REPEAT trial).

Note that full information on the approval of the study protocol must also be provided in the manuscript.

Clinical data

Policy information about clinical studies

All manuscripts should comply with the ICMJE guidelines for publication of clinical research and a completed CONSORT checklist must be included with all submissions.

Clinical trial registration	REPEAT trial (Repetitive Progenitor Cell Therapy in Advanced Chronic Heart Failure; NCT 01693042)
Study protocol	Assmus, B. et al. Improved outcome with repeated intracoronary injection of bone marrow-derived cells within a registry: rationale for the randomized outcome trial REPEAT. Eur. Heart J. 37, 1659–1666 (2016).
Data collection	Clinical data collection was conducted in accordance with the REPEAT trial study protocol.
Outcomes	REPEAT trial aimed the comparison of the effects of single versus repeated intracoronary application of bone-marrow cells on 2-year mortality in patients with chronic post-infarction heart failure. While bone marrow samples of 19 consecutive REPEAT patients were investigated in this study (all at baseline, before the first intracoronary application of bone marrow cells, irrespectively of the study randomization), the outcome data from the REPEAT trial were not relevant for the current manuscript.

Flow Cytometry

Plots

Confirm that:

- [x] The axis labels state the marker and fluorochrome used (e.g. CD4-FITC).
- [x] The axis scales are clearly visible. Include numbers along axes only for bottom left plot of group (a 'group' is an analysis of identical markers).
- [x] All plots are contour plots with outliers or pseudocolor plots.
- [x] A numerical value for number of cells or percentage (with statistics) is provided.

Methodology

Sample preparation	Mouse flow cytometry<br>Isolation of mouse bone marrow: For flow cytometric analysis, tibiae and femurs were collected, cleaned thoroughly to remove the adherent muscles. Cleaned bones were then crushed in ice cold PBS with mortar and pestle. Whole bone marrow was digested with collagenase II (420U; C2-22; Millipore) at 37°C for 20 minutes. After digestion, cells were washed two times with PBS and filtered through a 100 µm cell strainer (EASYstrainerTM 100 µm; Greiner Bio-One). The cell concentration was adjusted to 106/100µl in cell stain buffer (Biolegend).<br><br>Human bone marrow flow cytometry<br>100µl bone marrow were blocked with 2µl Fc Receptor Blocking Solution (Human TruStain FcX™; 422301; Biolegend) for 10 minutes at room temperature. BV-421-conjugated CD31 (303124; Biolegend; clone WM59), APC-Cy7-conjugated CD45 (368516; Biolegend; clone 2D1), FITC-conjugated Lineage cocktail 4 (562722; BD; clones RPA-2.10, HIT3a, RPA-T4, M-T701, HIT8a, B159, GA-R2 (HIR2)), APC-conjugated AC133 (130-090-826; Miltenyi Biotec), PE-Cy7-conjugated CD34 (343516; Biolegend; clone 581) and biotin-conjugated Endomucin (ab45772; abcam; clone TX18) were added to the bone marrow and incubated for 20 minutes. After staining BM was washed two times with cell stain buffer followed by a second incubation step with PE-conjugated Streptavidin (405204; Biolegend) for 20 minutes. Erythrocytes were lysed with 1x RBC lysis buffer (420301; Biolegend) for 10 minutes and washed two times with cell stain buffer. After adding 7AAD viability staining solution (420404; Biolegend), cells were measured on BD FACS Canto II flow cytometer and analysed using FlowJo (Version 10; FlowJo LLC).

Instrument	BD FACS Canto II flow cytometer
Software	FlowJo (Version 10, FlowJo LLC).
Cell population abundance	The specific cell populations are determined with respect to the input relative to expression analysis of EC and HSC markers having begun the analysis with at least 10^6 cells per sample and acquisition of at least 100,000 viable and single events .
Gating strategy	The initial gating involved exclusion of debris with FSC/SSC by excluding low FSC/SSC values (extreme lower left quadrant), followed by positive selection for live cells, with a subsequent gate applied to select for single cells while excluding doublets. With this population we utilized two-dimensional plots (pseudo-color or dot plot) created using biexponential transformation and sequential gating of EC and HSC cell subsets according to the established models using the antibodies described above. Gates were set on FMO controls.

☒ Tick this box to confirm that a figure exemplifying the gating strategy is provided in the Supplementary Information.
